# An approach for mixture testing and prioritization based on common kinetic groups

**DOI:** 10.1007/s00204-022-03264-8

**Published:** 2022-03-19

**Authors:** Albert Braeuning, Denise Bloch, Mawien Karaca, Carsten Kneuer, Stefanie Rotter, Tewes Tralau, Philip Marx-Stoelting

**Affiliations:** 1 Department of Food Safety, German Federal Institute for Risk Assessment, Max-Dohrn-Str. 8-10, 10589 Berlin, Germany; 2 Department of Pesticides Safety, German Federal Institute for Risk Assessment, Max-Dohrn-Str. 8-10, 10589 Berlin, Germany

**Keywords:** Mixtures, Risk assessment, Toxicokinetics, Grouping approach, Pesticides

## Abstract

In light of an ever-increasing exposure to chemicals, the topic of potential mixture toxicity has gained increased attention, particularly as the toxicological toolbox to address such questions has vastly improved. Routinely toxicological risk assessments will rely on the analysis of individual compounds with mixture effects being considered only in those specific cases where co-exposure is foreseeable, for example for pesticides or food contact materials. In the field of pesticides, active substances are summarized in so-called cumulative assessment groups (CAG) which are primarily based on their toxicodynamic properties, that is, respective target organs and mode of action (MoA). In this context, compounds causing toxicity by a similar MoA are assumed to follow a model of dose/concentration addition (DACA). However, the respective approach inherently falls short of addressing cases where there are dissimilar or independent MoAs resulting in wider toxicokinetic effects. Yet, the latter are often the underlying cause when effects deviate from the DACA model. In the present manuscript, we therefore suggest additionally to consider toxicokinetic effects (especially related to xenobiotic metabolism and transporter interaction) for the grouping of substances to predict mixture toxicity. In line with the concept of MoA-based CAGs, we propose common kinetics groups (CKGs) as an additional tool for grouping of chemicals and mixture prioritization. Fundamentals of the CKG concept are discussed, along with challenges for its implementation, and methodological approaches and examples are explored.

## Introduction

Toxicological risk assessments are mainly based on the toxicological characterization and assessment of individual compounds with potential mixture effects only being considered in selected cases such as pesticides (Stein et al. 2014) or in other cases of foreseeable co-exposure (e.g., food contact materials). The latter is a precondition for mixture effects to occur, as are sufficiently high doses. It is for this reason that incidences of known mixture toxicity are generally low and that single substance assessments are in most cases considered sufficiently conservative (Herzler et al. 2021). Yet, the ever-increasing use of and exposure to chemicals have led to regained regulatory awareness of potential mixture effects (Tralau et al. 2021). This is particularly the case for chemical substances regulated under REACH (EU legislation on the Registration, Evaluation and Authorization of Chemicals), but has also stimulated conceptual debates for those regulatory domains where potential mixture effects already are already considered to some extent, such as pesticides.

The respective assessments often follow component-based approaches, which require information of the substances mode of action (MoA). In the field of pesticides, active substances are summarized in so-called cumulative assessment groups (CAG). For the sake of feasibility and handling, this approach is primarily based on toxicodynamic properties, that is, respective target organs and MoAs. Compounds sharing a similar MoA can then be assessed using the dose/concentration addition (DACA) model. However, such toxicodynamic-based approaches inherently fall short of addressing wider toxicokinetic effects caused for example by dissimilar or independent MoAs. It is often these latter cases where the effects of the corresponding mixtures are at risk of being underestimated as they can significantly deviate from DACA.

In the area of pharmaceutical drug safety and regulation where the investigation of drug–drug interactions (DDIs) is mandatory, possible toxicokinetic interactions therefore are a key focus during the approval of new active substances for human or veterinary therapy (Pelkonen et al. 2020). Indeed, instructions on the study of interactions at the level of absorption, distribution, metabolism and excretion (ADME) dominate the current guideline on the investigation of drug interactions issued by the European Medicines Agency (EMA 2012). Numerous pharmacological interactions, often based on interference with the cytochrome P450 (CYP)-mediated phase I metabolism of active substances, have been published and discussed in depth elsewhere (Tralau and Luch 2012, 2013). Similarly, various DDIs resulting from interference at the level of transmembrane transport have been identified and discussed with regard to their practical consequences for regulatory assessment by the International Transporter Consortium (Tweedie et al. 2013).

Although DDI is roughly the pharmaceutical equivalent to mixture toxicity, there are some important differences when it comes to the assessment of chemicals. First, drugs usually are designed for and take effect on specific molecular targets with an established MoA and well-investigated pharmacokinetics. Contrastingly, chemicals not only tend to be more promiscuous and less specific in their toxicity, but also often lack data regarding their MoA and kinetic behavior. Second, patients are usually intendedly exposed to a limited number of drugs at pharmacologically relevant dose levels at a time, while consumer exposure to chemicals often occurs unintendedly, e.g., via residues or contaminants in food and drinking water. Whereas the less systematic exposure of consumers undoubtedly complicates targeted assessment, it also acts as a kind of gatekeeper with regard to potential mixture effects, as for these to occur requires co-exposure at doses high enough to yield an effect. Generally, consumer exposure to chemicals occurs at low levels, mostly below the respective toxicological reference values like the acceptable daily intake (ADI) or the tolerable daily intake (TDI).

However, with about 22,000 compounds currently registered as industrial chemicals under REACH, the number of chemical combinations available for exposure is almost infinite. Add to this some 470 and 150 active substances approved for use in plant protection products and biocides in the EU, and there is unarguably a need to develop strategies for the identification and prioritization of potentially critical mixtures even in the absence of an immediate concern (Tralau et al. 2021). Given the large chemical space, any wider regulatory strategy on mixture toxicity will in the end have to settle for a two-tier system. First, there needs to be a filtering step to narrow down and define the number of substance combinations to be looked at. Ideally, this should be done with a strong focus on exposure, which can, if needed, be complemented by considerations regarding hazard potential (Herzler et al. 2021; Tralau et al. 2021). The actual assessment then follows in a second step that will strongly rely on the toxicological evaluation of the potential effects and in that context grouping and prioritization.

In this step, mixtures, which might exert effects that deviate from the general assumption of dose/concentration addition (DACA), are of specific interest. Such mixtures of concern might be identified by grouping chemicals based on their toxicological properties, as, e.g., already established for pesticides. Here, so-called cumulative assessment groups (CAG) are used to classify active compounds (EFSA 2021). For practical reasons, the CAG concept has historically put a strong focus on toxicodynamics. Although CAG formation thus usually omits broader toxicokinetic considerations, it can at times comprise kinetic aspects specifically linked to the respective adverse outcome at the organ level. For example, transport and metabolism of thyroid hormones were parameters used when compiling a CAG for the thyroid gland, as was induction of phase I biotransformation enzymes for a CAG for liver (Nielsen et al. 2012). Notably, a common target organ and/or MoA is a precondition for any CAG-based approach. Such grouping approaches in combination with exposure or risk considerations are frequently used as a starting point for the selection and prioritization of chemicals for mixture toxicity testing and assessment (OECD 2018). One prominent example for this is the EU-funded project “EuroMix”, where a number of tools and methodologies for mixture risk assessment have been developed and evaluated. The project strongly relied on adverse outcome pathways (AOP) and thus in turn substance interactions such as for example the ability of a test compound to interact with certain molecular targets and exert toxicological effects. As a consequence mixture selection in “EuroMix” was essentially based on the toxicodynamic properties of the individual test chemicals (Alarcan et al. 2021; Beronius et al. 2020). This is also further substantiated in the recent European Food Safety Authority (EFSA) Mixtox Guidance (EFSA 2021).

In a comprehensive systematic review, Martin et al. (2021) recently analyzed publicly available data on mixture effects. The authors concluded that while DACA appeared to be sufficiently protective for most mixtures, the potential for non-additive, especially synergistic effects, should not be disregarded, particularly for some classes of chemicals. They also confirmed that synergistic effects are often linked to interferences at a toxicokinetic level, for example to interactions affecting the corresponding compounds’ ADME properties. This observation is well in line with what we have seen in our own regulatory work and research projects as well as with what has been reported by others (Cedergreen 2014).

Recent findings by Lasch et al. (2021) for example show that triglyceride accumulation caused by the triazole-class fungicides propiconazole, tebuconazole and difenconazole in HepaRG cells is exacerbated by the presence of the non-steatotic pyrrole-class fungicidal compound fludioxonil. This effect is related to an interference of fludioxonil with the CYP-mediated metabolism of the triazoles (Lasch et al. 2021). In line with this, studies in vivo also showed deviation from DACA for the combination of the three closely related azole fungicides cyproconazole, epoxiconazole, and prochloraz (Schmidt et al. 2016). The data presented in the aforementioned papers highlight the relevance of toxicokinetic interference as a decisive cause of mixture effects deviating from DACA, both in vitro and in vivo. In addition, the observation that combinations of a steatotic substance with a non-steatotic substance amplifies the observed steatotic effect in liver cells (Lasch et al. 2021) highlights possible limitations of a prioritization and grouping strategy, which predominantly relies on the constituents’ toxicodynamic properties.

**Fig.1:**
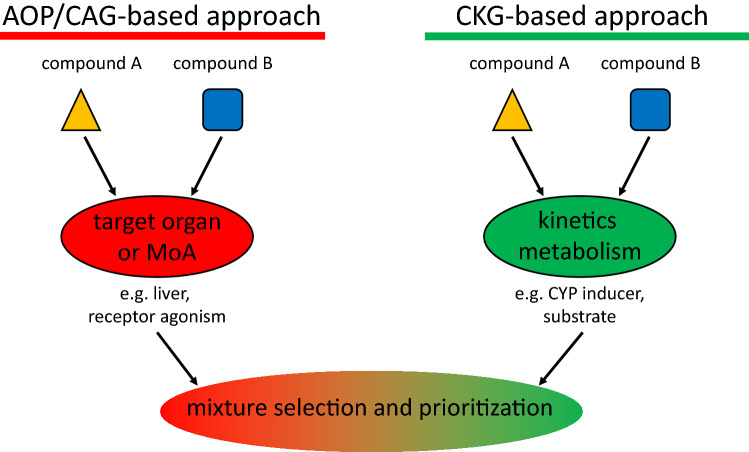
Comparison of common assessment group (CAG) and common kinetic group (CKG) approaches. Adverse outcome pathway (AOP)- and CAG-based approaches focus on toxicodynamic properties (target organ and/or mode of action (MoA), while the CKG-based approach centers on toxicokinetics (e.g., the ability of a compound to interfere with drug-metabolizing enzymes

Undoubtedly, CAG- and AOP-based approaches are useful as grouping tools and for the selection and prioritization of chemical mixtures. Given the data routinely at hand, they are also often a practical way forward. This should not lead to an underestimation of effects though. With the concept of chemical grouping based on toxicokinetic similarities not being entirely new (OECD 2018), it therefore appears timely to complement the existing strategies for mixture categorization with more comprehensive toxicokinetic considerations. Respective data should cover the induction and inhibition of key enzymes for phase I and phase II metabolism, as well as interferences related to transport processes. We therefore recently proposed developing a novel grouping system based on common kinetic groups (Braeuning and Marx-Stoelting 2021). A schematic overview of respective AOP/CAG- and CKG-based strategies is presented in Fig. 1. A CKG concept may be used to identify, select and prioritize compounds and mixtures thereof with toxic effects potentially exceeding DACA. Here, we substantiate the concept of CKG and discuss important issues to consider when establishing CKGs. In addition, examples for CKG grouping are presented.

## How to construct a CKG?

**Fig.2:**
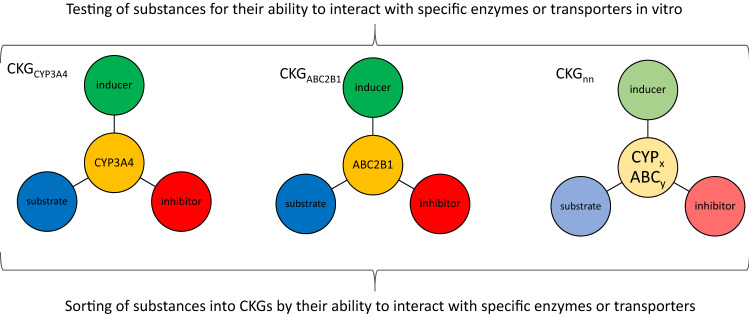
How to build CKGs: especially interactions with drug-metabolizing enzymes such as cytochrome P450 (CYP) enzymes or with transporters such as the ATP-binding cassette (ABC) transporters play a role in drug–drug interactions (DDIs). Some enzymes are most prominent here, for example CYP3A4. Hence, in vitro testing as performed in the field of pharmaceuticals may form the basis of CKG construction

In principle, toxicokinetic interactions of compounds may involve all aspects of toxicokinetics, classically categorized within the ADME concept. Not all of these aspects are considered equally relevant in the fields of pharmacology or toxicology: for example, high concentrations of some pharmaceutical compounds are capable of displacing others from plasma protein binding, thus altering their pharmacokinetic properties. This effect is not considered a likely cause of interaction in the case of exposure to comparably low doses of multiple environmental chemicals or pesticide residues in food. By contrast, interference of a compound (or several similar compounds for that matter) with specific metabolic enzymes or transport proteins may play a role, sufficient potency provided. Especially, the induction or inhibition of enzymes or transporters involved in biotransformation needs to be considered in this context. The Transformer database currently lists 4407, 431 and 1158 of such interactions with phase I enzymes, phase II enzymes and drug transporters, respectively (Hoffmann et al. 2014). Construction of CKGs should therefore primarily focus on interactions with these proteins (Fig. 2).

Xenobiotic-metabolizing enzymes from the CYP superfamily are among the most important enzymes when it comes to phase I metabolism of drugs and environmental chemicals. The isoforms 3A4/5, 2D6, 2C9, 1A2, 2B6, 2C19, 2C8, 2A6, 2E1, and 2J2 are frequently involved in the metabolism of drug molecules (Li 2021) with CYP3A4 being the oxygenase involved most frequently (Huang et al. 2008). Interference with CYPs therefore has been a frequent underlying cause for kinetic interactions such as the aforementioned effects observed by Lasch et al. (2021) for the three triazole fungicides. For recent overviews see, e.g., Pelkonen et al. (2020) and Tralau and Luch (2012, 2013). Likewise, frequently affected are systems involved in the active uptake or excretion of xenobiotics as exemplified by the high number of known drug-transporter interactions (Hoffmann et al. 2014). The potential of transporters to affect significantly a chemical’s ADME fate at all levels has been demonstrated repeatedly. The main players include MRPs (multidrug resistance-related proteins), MDRs (multidrug-resistance proteins), OATPs (organic anion-transporting peptides), OATs (organic anion transporters), OCTs (organic cation transporters), and others at the intestinal, hepatic, renal and/or other protective barriers (Fu et al. 2021). Especially, the ATP-binding cassette (ABC) transport protein family is one of the most important systems for the efflux of chemicals from cells. Hence, inhibition and induction of transporters are frequent mechanisms relevant for toxicokinetic interactions (FDA 2020; Stevenson et al. 2006).

Having said that, it should, however, be noted that other groups of genes might be relevant for compound interactions as well, for example effects on DNA repair enzymes in the case of exposure to genotoxic compounds.

## Information source for CKG assignment

Methodologically, both in vitro and *in silico* assays are suited to be the primary source of data for sorting substances into CKGs, of course considering the general limitations of such approaches. In vivo information, e.g., on biomarkers for effects on biotransformation and transport activity can provide supporting information, but may not be available in many cases.

Currently, EU chemical legislation REACH does not require the generation of any specific information on the ADME properties of a chemical at any tonnage. Nevertheless, summaries of existing toxikokinetic data for some well-researched chemicals are present in REACH dossiers and made available through the European Chemicals Agency (ECHA) dissemination website. Unfortunately, this information is mostly limited to data from OECD TG 417 with the focus on the absorption, distribution and excretion of a radiolabel in laboratory animals. In some cases, biotransformation products are identified and quantified in excreta, providing some additional information on the likely metabolic pathway in rodents. This is also the typical level of information available for biocides. Meanwhile for pesticides, data on comparative in vitro biotransformation is required since 2013 (EC 2013). However, while this allows a better understanding of the respective compound’s metabolism, identification of the enzymes catalyzing the biotransformation is not a requirement. Information on the enzymes involved therefore is limited to what data are publicly available, including potential inhibitors or interactions with cellular transport.

As enzyme and transporter induction is frequently linked to adaptive and adverse toxicodynamic outcomes, some indications on these aspects may in fact be gained from mechanistic studies (if performed). Indeed, a draft test guideline named “Determination of Cytochrome P450 (CYP) enzyme activity induction using differentiated human hepatic cells” has been developed by the OECD (2019). However, this test guideline has not yet been finalized following consultation in 2019. For a start, CKG formation for chemicals, including biocides and pesticides, will thus have to rely on the limited data publicly available, generation of further data pending.

In the field of pharmaceuticals, in vitro experiments and clinical pharmacokinetic data may give the first mechanistic information on DDIs and the need and design of further studies. For detection of such interactions, a number of methods exist (Wienkers and Heath 2005). For example, reporter gene assays indicate whether a substance is able to induce genes for a certain CYPs or transporters (Willson and Kliewer 2002). Moreover, in vitro batteries detecting substrates or inhibitors of CYPs are applied to detect potential DDIs. Fu et al. (2021) have recently summarized the portfolio of existing in vitro methods for identifying and characterizing transporter substrates and inhibitors. Table 1 provides an overview of some of the in vitro methods recommended for the investigation of pharmacokinetic drug interactions and is based on the guidelines of European and US regulatory agencies (EMA 2012; FDA 2020). For pharmaceuticals, the methodology needed for assigning chemicals to CKGs therefore in large has already been explored and established. In principle, these tools would also be applicable to chemicals, therefore omitting the need of major development of new tests.

**Table 1:** Overview of recommended in vitro methods for the investigation of pharmacokinetic drug interactions by the European Medicines Agency (EMA) and the US Food and Drug Administration (FDA)

	EMA Guideline on the investigation of drug interactions	FDA In vitro drug interaction studies—cytochrome P450 enzyme- and transporter-mediated drug interactions—Guidance for Industry
Involvement of transporters in drug absorption	- Transport studies in Caco-2 cells in both directions - If ratio of basolateral (B) to apical (A) and A to B permeation is > 2 or < 0.5→ involvement of an efflux transporter or an active uptake transporter is suggested - Presence of expression of transporters (P-gp, BCRP, PEPT-1) should be investigated→determination of permeability ratios of positive controls - Identification of involved transporters: investigation of permeability ratio with and without selective inhibitors in Caco-2 cells	- Bi-directional transport assays with cell-based systems - Calculation of the apparent permeability (P_app_) in both direction A→B (absorption) and B→A (efflux) and calculation of the efflux ratio - Involvement of ABC transporters (BCRP, P-gp): Caco-2 cells, membrane vesicles, knock-out/down cells, transfected cells
Involvement of hepatic metabolizing enzymes	- Human liver microsomes, hepatocytes, recombinant cells expressing human liver enzymes, liver S9 fractions, etc. - CYP and UGT enzymes are present in all systems - Cytosolic enzymes are present in S9 fractions and hepatocytes - Identification of involved enzymes, when using multi-enzyme systems: adding specific enzyme inhibitors (chemicals/drugs or antibodies) - If no specific inhibitor is available: use of in vitro systems where only the particular enzyme is expressed	- Subcellular human liver tissue fractions such as human liver microsomes, liver S9 fractions, recombinant human CYP enzymes, and human liver tissues, including freshly prepared hepatocytes and cryopreserved hepatocytes - Identification of involved CYP enzymes by using both of the following methods: 1. Specific enzyme inhibitors (chemicals/drugs or antibodies) in human liver microsomes or hepatocytes 2. Individual human recombinant CYP enzymes
Investigation of CYP enzyme inhibition or induction	- CYP inhibition studies: human liver microsomes, hepatocytes, or other cells expressing the investigated enzyme: metabolism of a specific substrate→monitoring enzyme activity and investigation of the inhibition constant (KI) - CYP induction and down-regulation studies: cultured hepatocytes (fresh or cryopreserved)→ activation of nuclear receptors (Ah-receptor), enzyme down-regulation - Minimally derived hepatocyte line (e.g., HepaRG), nuclear receptor binding assays, reporter gene assay as supportive data	- CYP inhibition studies: human liver microsomes, microsomes obtained from recombinant CYP-expression systems, or hepatocytes→analytical assay to measure the formation of a substrates metabolite and calculation of the KI value - CYP induction studies: fresh human hepatocytes, immortalized hepatic cell lines→mRNA levels and/or enzyme activity levels
Determination of involved transporters affecting drug disposition	- Identification of specific transporters involved in uptake or efflux process in drug disposition: 1.(over-)expressing of transporter in vector systems and comparing to normal vectors 2.Using selective inhibitors for specific transporters 3.Inhibition of transporter expression (knocking out of genes, silencing mRNA)	- Uptake assays with cell-based systems for substrate or inhibitory properties - Transfected cell lines→comparison with drug uptake in parental or empty vector-transfected cell line

Several large databases provide information about possible pharmacokinetic interactions. Examples are the Transformer—Metabolism of Xenobiotics database (Hoffmann et al. 2014), the “SUPERCYP” database (Preissner et al. 2009), or the Kardiolab database for CYP interactions (https://www.kardiolab.ch/CYP450_2JSI.html). In addition, predictive *in silico* models have been developed to generate some understanding of the likely metabolic pathways for a chemical substance as well as potential biotransformation enzymes and transporters involved.

More recently, the use of biomarkers for detecting the inhibition or induction of biotransformation enzymes and transporters in vivo, i.e., in humans and animals, has been discussed intensively (Chu et al. 2018). This latter approach allows obtaining evidence for physiologically relevant repression or induction of the respective pathway in vivo and at the same time allows describing rather than model the respective dose–response relationship. Notably, biomarker measurements could be included in existing in vivo protocols, extending their predictive value from single substance toxicology to potential mixture toxicity.

## Complexity versus clarity

### Types of substances to be included in each CKG

Mechanistically, different groups of compounds will have to be considered within a CKG. The first group of compounds is constituted by substrates of the respective metabolic enzyme or transport protein. Adding more complexity to the system, the CKG will also have to comprise inducers as well as inhibitors of the respective enzyme or transporter, thus merging different molecular MoAs (see also Fig. 2 for illustration). This approach is similar to what is already done for pharmaceutical compounds; e.g., within the databases mentioned above.

### Which CKGs should be considered?

The human genome encodes almost 60 different CYP enzymes, of which especially members of families 1–3 are active in xenobiotic metabolism (Zanger et al. 2014). When considering CKG as an extension to the existing CAG approach, substances affecting an individual CYP enzyme like CYP3A4 should consequently be summarized in one CKG. Comparable considerations apply to the family of ABC transporters, which comprises 49 members within the human genome (Vasiliou et al. 2009). Initially the number of CKGs would practically be limited to those phase I and phase II enzymes as well as transporters, for which toxicokinetic interactions have been observed or at least predicted in the past. While the knowledge on DDIs and interactions described in the scientific literature and/or included in the EMA and FDA guidelines may serve as a starting point, existing experience relies largely on DDIs. Therefore, additional enzymes/transporters may need to be taken into consideration for chemicals, while others frequently relevant to drugs might turn out to be less important.

### Complexity of CKG assignment

Many compounds display a complex ADME pattern involving multiple steps and enzymes. Without further prioritization, displaying the full complexity of in vivo metabolism in a CKG system would thus most likely lead to network too complex as to allow straightforward regulatory conclusions on potential mixture toxicity. For this reason, mapping and filtering the metabolic pathways for those most relevant will be a key task for establishing CKG-based assessment. In this context, also genetic polymorphisms in ADME genes might be necessary to consider in some cases.

Inhibition of CYPs is mainly caused by direct interaction of the respective compounds with the enzyme’s active center and as such accessible to testing or, in case of a crystal structure, modeling. Likewise, induction of CYP activity is frequently related to nuclear receptor-mediated mechanisms the activity of which can be interrogated using reporter systems. Finally, protein stabilization can play some role in xenobiotic-mediated alterations of CYP activities, e.g., for CYP2E1 (Gonzalez 2007; Gonzalez et al. 1991). However, while again this can be tested for the characteristic of being a CYP substrate, inhibitor or inducer is often not limited to a single isoform. Many compounds will therefore be part of more than one CYP- or transporter-related CKG.

For ABC transporters, the processes of transport, inhibition, and induction can be interlinked. Substrates may act as competitive inhibitors when present at higher concentrations (Epel et al. 2008). For example, the P-gp (ABCB1) substrate verapamil inhibits the transport of other substrates by rapid diffusion back into the cell from which it is then effluxed again, thus competitively claiming the entire transport capacity (Litman et al. 2001). Somewhat comparable to CYP enzyme induction by xenobiotics via protein stabilization, transport inhibition due to chronic capacity overload can lead to the induction of more transporters. Thus, inhibitors or competitive substrates can become ABC protein inducers. The respective induction can be a specific response to high concentrations of one single substrate or inhibitor (Lemma et al. 2006), but has also been shown to occur upon exposure to lower doses of a variety of substances (Kurelec 1995). In the latter case, induction is a response to a generally high workload that cannot be traced back to specific substances (Elmeliegy et al. 2020). Some ABC transporters, e.g., P-gp, possess unspecific binding domains and transport a variety of structurally different compounds. Others, e.g., MRP2, specialize on ionized and conjugated chemicals (Bard 2000; Borst and Elferink 2002). Hence, toxicokinetic effects on metabolism, affecting CYP reactions and/or phase II conjugation reactions, can be interlinked with effects on ABC transport.

### Challenges by multi-compound mixtures

Several scenarios have to be considered when addressing toxicokinetic effects on transporters or xenobiotic-metabolizing enzymes by multi-compound mixtures. First, what if several substrates are present in a mixture? As long as the capacity of an enzyme or transporter is far from saturation, all substrates are converted or transported and influences on other substrates also present at low concentrations are expected to be minor. In this context, information on exposure levels will certainly be helpful to judge on the relevance and/or likelihood of mixture effects. When approaching saturation, however, it can reasonably be assumed that substrates with higher binding site affinity are processed first, while all others accumulate within the cell.

Second, what if several inhibitors are present in a mixture? Competitive inhibitors are often substrates characterized by high binding site affinity but slow rates of catalysis or transport. Therefore, they slow down the turnover of other substrates and competitive inhibitors, but will eventually be eliminated from the cell. That is, unless their net uptake is faster than their metabolism or efflux. Non-competitive ATPase inhibitors can lead to long-term inactivation of ABC transporters (Lentz et al. 2000; Litman et al. 2001). They will even prevent slow efflux and may only be counteracted by the induction of higher levels of transporters. Thus, even mixtures of inhibitors may display increased effects, for example if a toxic competitive inhibitor co-occurs with a non-competitive or ATPase inhibitor.

Third, what if substrates and inhibitors are present in a mixture? The observed effects may mirror the example of the presence of a toxic competitive and another inhibitor, since competitive inhibitors are often, ultimately, substrates themselves. Where toxic substrates are present in a mixture with an inhibitor, increased effects may be observed.

Fourth, what if inhibitors and inducers are present in a mixture? Long-term inhibition may eventually lead to the induction of the inhibited proteins, as for example reported for transporters (Kurelec 1995). Presumably, the faster the induction of new transporters, the less relevant the influence of inhibitors becomes. However, since ABC transport is energy-dependent this may still affect the cell by draining its energy reserves (Litman et al. 2001; Stevenson et al. 2006).

Fifth, how can metabolism and transport interact? Higher metabolism rates of one component of a mixture may lead to that substance being removed from the cell more efficiently. Be it either by improved binding site affinity or by decreased re-uptake across the lipid bi-layer membrane. Hence, substances competing for metabolic enzymes may indirectly compete for efflux. Potentially, this could augment adverse effects by more slowly metabolized and toxic ABC transporter substrates.

### Approaches to reduce complexity

In principle, two strategies can be used to reduce the aforethought complexity. First, addition of (semi-)quantitative aspects (e.g., “strong inducer”, “weak inhibitor”, “main metabolic pathway”) will help to filter for potentially critical interactions within a high number of CKG entries. This way, any further differentiation for competitive or non-competitive inhibition also will become unnecessary, as the inclusion into a CKG will be primarily based on the effect, not the specific mechanism.

Second, it could be discussed whether meaningful cutoffs for inclusion in a certain CKG can be defined (e.g., “at least 10% of compound metabolized by CYP3A4”, “inhibition only at concentrations exceeding X µM”). This also would result in a considerable reduction of the number entries of a CKG. As mentioned before testing systems, e.g., for CYP activities, are well established hence allowing for this kind of selection based on compound screening.

For drugs, the biopharmaceutical classification system (BCS) has been introduced to classify drug candidates in accordance to the likelihood that they will display variable toxicokinetic behavior through certain types of interaction (ICH 2021). This allows focusing on those substances and pathways with higher likelihood of effect. For example, substrate studies with MDR1 and BCRP (Breast cancer resistance protein) are recommended for BCS class 1 drugs with low oral bioavailability as well as for low solubility and high permeability class 2 drugs, while high solubility/low permeability BCS class 3 drugs qualify for evaluation for absorptive transport by OATP1B1/1B3 and renal clearance-related transport by OAT1, OAT3 and OCT2 is regarded relevant for class 4 low solubility/permeability drugs (Fu et al. 2021). In analogy to the BCS, a chemical that is a substrate of a biotransformation enzyme and/or transporter, but both unlikely to be significantly affected in terms of its kinetics (*C*_max_, area under the curve (AUC)) or to significantly inhibit or induce this pathway, may not qualify for a CKG. Notably, criteria have been defined by the leading drug authorities to decide whether the inhibitor or substrate properties determined for a drug candidate in vitro warrants further follow-up in vivo DDI studies. The quantitative cutoff values set depend on the individual activity and potency. Such decision criteria could be translated into chemical safety assessment. For this purpose, the maximum concentration of the substance in the gut or other compartments could be estimated from an established reference dose.

With regard to transporters, substrates and inhibitors may be defined based on their net effect on specific transporters. Thus substrates with a high net uptake by passive diffusion, such as the P-glycoprotein (P-gp) substrate verapamil, should be effectively regarded as inhibitors as they will effectively outcompete any other effect be it inhibitory or activating. The identification of potential inducers should be limited to substances, which have been demonstrated to cause transporter induction in relevant test systems. In addition, for the sake of reducing complexity the potential role of inhibitors and substrates as unspecific inducers may probably be disregarded. In doing so, one allows for worst-case scenarios where transport inhibition leads to increased cellular accumulation. To account for the efflux of metabolites by transporters, they should be included in the list of transporter substrates wherever such information is available.

Mathematical models have been developed to quantitatively predict the complex interplay between inducers, inhibitors and substrates of one or more relevant transporters. In some cases, such models have shown remarkable predictivity for certain combinations of drugs and transport pathways. For example, when the interaction of rosuvastatin with the drugs rifampicin, asunaprevir, and velpatasvir mediated by hepatic OATP1B1/3 and intestinal breast cancer resistance protein (BCRP) was modeled as the ratio of the area under the curve (AUC), predicted values were within a 1.5- to 2-fold range of what was measured in vivo (Sane et al. 2020). However, a recent comprehensive analysis by Taskar et al. (2020) still identified a large number of practical challenges with regard to general applicability of such complex physiologically-based toxicokinetic (PBTK) models for predicting transporter/enzyme mediated DDIs. Nevertheless, such models may be instrumental to further develop and validate qualitative decision criteria as those discussed above.

## Examples for CKGs: CYP3A4/5 and ABCC2

In the following, we will focus on two prototypical toxicokinetic interactions as representative examples for ADME-relevant proteins and the construction of CKGs: the CYP enzyme CYP3A4 as a representative of phase I metabolism, and the transporter ABCC2 (also termed MRP2) as a representative of the ABC transporter family. By referring to these two prominent examples. we show how CKGs may be created and composed.

Based on the above considerations, two draft candidate CKGs are presented in Tables 2 and 3, respectively. These are by far not complete and just given to illustrate how a CKG could look like. Table 2 shows the membership of selected pesticidal active compounds in a CKG for CYP3A4, while an exemplary suggestion of substance classification for compounds being substrates of or activating the MRP2 transporter is provided in Table 3.

**Table 2:** Draft CKG for CYP3A4/5. The CKG was compiled to illustrate how a CKG could look like in contrast to a CAG

Substance	Substrate	Inhibitor	Inducer	Reference(s)
Epoxiconazol	()	()	Yes	Heise et al. (2015), Zahn et al. (2018)
Difenoconazol	Yes	()	Yes	Lasch et al. (2020)
Propiconazole	()	()	Yes	Knebel et al. (2019)
Fludioxonil	()	Yes	No	Lasch et al. (2020)
Cyproconazol	()	()	Yes	Zahn et al. (2018)
Tebuconazol	()	()	yes	Knebel et al. (2019)

Considering the multitude of chemical substances and the limited knowledge on toxicokinetics (including CYP-dependent metabolism) of many of those compounds, the CKG is certainly not complete and information on some abilities of the substances is missing. For a recent review on CYP-inducing azoles see Marx-Stoelting et al. (2020)

**Table 3:** Draft CKG for ABCC2

Substance	Substrate	Inhibitor	Inducer	Reference(s)
GSH conjugates of metals, e.g., As, Sn, Ag, Cd	X			Suzuki and Sugiyama (2002) Kala et al. (2000)
Glucuronide conjugates of drugs, e.g., Diclofenac Acetaminophen	X			Seitz et al. (1998) Xiong et al. (2000)
Non-conjugated drugs, e.g., Etoposide Vincristine Doxorubicin Cisplatin	X			Cui et al. (1999)
Ochratoxin A	X			O'Brien and Dietrich (2005)
Rifampicin			X	Fromm et al. (2000)
Curcumin		X		Ge et al. (2016)
Phenobarbital		X		Xiong et al. (2002)

The CKG was compiled to illustrate how a CKG could look like in contrast to a CAG. Considering the multitude of chemical substances and the limited knowledge on toxicokinetics (including transport) of many of those compounds, the CKG is certainly not complete

The information depicted in Tables 2 and 3 should, of course, not be perceived as a complete CKG. Instead, a number of chemicals have been picked, for which information on CYP or transporter interaction is available. These are exemplarily shown to illustrate how substrates, inducers and inhibitors could be included into a CKG, and how available information can be utilized to predict interactions based on toxicokinetic interference.

Table 2 shows that various triazole-class fungicides are inducers of CYP3A4 activity, which is mechanistically based on pregnane-X-receptor (PXR) and constitutive androstane receptor (CAR) activation by the compounds, followed by subsequent transcriptional activation of the *CYP3A4* gene (Braeuning and Marx-Stoelting 2021). Fludioxonil, by contrast, inhibits CYP3A4 activity. Taken together, this information can be used to predict a potential interaction between fludioxonil and, e.g., difenoconazol, leading to effects potentially deviating from the DACA concept. Existence of this particular interaction has been demonstrated by Lasch et al. (2020). Similarly, Table 3 shows selected substrates, inhibitors, and inducers of the transport protein ABCC2. The simultaneous presence of, e.g., a substrate and an inhibitor may give rise to the assumption that interactions could occur, which lead to effects beyond what is covered by the DACA concept.

## Conclusion

This work has explored and exemplified how the basically toxicodynamic approach of the CAG and AOP concepts can be complemented by toxicokinetics-centered CKG. Notably both concepts should not be regarded as something fundamentally different or exclusionary as molecular interactions with cellular structures such as enzymes and receptors are underlying mechanisms of many toxicokinetic interactions. Ultimately, considering the toxicodynamic as well as toxicokinetic aspects of chemical mixtures in an integrated manner therefore will not only help mixture assessment in general, but appears a necessary precondition for singling out those mixtures that hazard-wise have the greatest potential for impacting consumer safety.
